# Supplementary material for: Why Do These Yeasts Smell So Good? Volatile Organic Compounds (VOCs) Produced by Malassezia Species in the Exponential and Stationary Growth Phases
Source: Molecules. 2023 Mar 14;28(6):2620. doi: 10.3390/molecules28062620 (PMC10056951; doi:10.3390/molecules28062620)
Supplement: Supplementary file 1 [file molecules-28-02620-s001.zip › molecules-2226164-supplementary.pdf]

Supplementary table S1. VOCs released by control (media without yeast).

No.	Compound	CAS	RI	Ret. Time
1	Methanol	67-56-1	382	1.45
2	Ethanol	64-17-5	448	1.51
3	Propan-2-one	67-64-1	500	1.60
4	Dimethyl sulfide	75-18-3	514	1.68
5	Acetohydrazide	1068-57-1		1.82
6	2-methylpentane	107-83-5	559	1.73
7	Hex-5-yn-1-ol	928-90-5		1.87
8	2-methylsulfonylacetic acid	2516-97-4		1.94
9	3-methylpentane	96-14-0	576	1.76
10	Ethenyl acetate	108-05-4	562	1.97
11	2-ethyloxetane			1.35
12	2-methylfuran	534-22-5	599	2.07
13	Oxolane	109-99-9	624	2.24
14	3-methylbutanal	590-86-3	669	2.47
15	2-methylbutanal	96-17-3	657	2.56
16	hept-1-ene	592-76-7	684	2.85
17	Pentanal	110-62-3	702	2.92
18	3,4-dimethyl-2,5-dihydrofuran	53720-72-2		3.42
19	Dimethyl disulfide	624-92-0	733	3.60
20	oct-1-ene	111-66-0	792	4.42
21	1,1,2,2-tetrachloroethene	127-18-4	806	4.80
22	Furan-3-carbaldehyde	498-60-2	846	5.31
23	(*E*)-hex-2-enal	6728-26-3	873	5.79
24	2,3-dimethylheptane	3074-71-3	859	5.90
25	6-methyloct-1-ene	13151-10-5		6.06
26	4-methyloctane	2216-34-4	864	6.10
27	Furan-2-ylmethanol	98-00-0	848	6.32
28	Heptan-2-one	110-43-0	890	6.81
29	2-butylfuran	4466-24-4	897	6.87
30	1-methylsulfanylpentane	1741-83-9	918	7.60
31	Unknown			7.72
32	Oxolan-2-one (Butyrolactone)	96-48-0	891	7.77
33	4-methylcyclohexan-1-ol	589-91-3	926	7.85
34	(3*E*)-nona-1,3-diene	56700-77-7	924	7.90
35	1-prop-2-enylcyclohexene	13511-13-2		7.97
36	Propylcyclohexane	1678-92-8	937	8.05
37	Butylcyclopentane	2040-95-1	941	8.18
38	3-propylcyclohexene	3983-06-0		8.50
39	Propan-2-ylidenecyclohexane	5749-72-4		8.51

40	Unknown			8.60
41	Unknown			8.67
42	Unknown			8.84
43	(*Z*)-hept-2-enal	57266-86-1	951	8.90
44	Benzaldehyde	100-52-7	960	9.06
45	2,2,6-Trimethyloctane	62016-28-8	964	9.13
46	2-methylnonane	871-83-0	970	9.27
47	1-(cyclohexen-1-yl)ethanol	3197-68-0		9.34
48	2,2,3,5-Tetramethylheptane	61868-42-6		9.60
49	2,5,6-Trimethyldecane	62108-23-0		9.73
50	1-octen-3-ol	3391-86-4	977	9.80
51	7-methyloct-3-yne	37050-06-9		9.91
52	Prop-2-enyl 2-ethylbutanoate	7493-69-8		9.98
53	(*E*)-2-ethylhex-2-en-1-ol	50639-00-4		10.06
54	3-pentylfuran	6177-84-0		10.91
55	Unknown			11.03
56	2,5-dimethylnonane	17302-27-1		11.18
57	4-methyldecane	2847-72-5	1060	11.52
58	Unknown			11.57
59	2,2,7,7-tetramethyloctane	1071-31-4		11.83
60	2,3,6,7-tetramethyloctane	52670-34-5		11.97
61	Octa-3,5-dien-2-ol	69668-82-2	1039	12.10
62	2,5,9-trimethyldecane	62108-22-9		12.17
63	3,4-Dimethyl-2-cyclohexen-1-one			12.66
64	5-ethyl-2,2,3-trimethylheptane	62199-06-8		12.77
65	(E)-oct-2-enal	2548-87-0	1055	12.86
66	2,4,6-trimethyldecane	62108-27-4		12.97
67	4-methyldecane	2847-72-5	1060	13.07
68	2-methyldecane	6975-98-0	1064	13.20
69	Unknown			13.36
70	Octan-1-ol	111-87-5	1068	13.47
71	2,3,5-trimethyldecane	62238-11-3		13.69
72	Unknown			13.76
73	2-ethyldodecan-1-ol	19780-33-7		13.87
74	2,6,8-trimethyldecane	62108-26-3	1104	14.17
75	2-hexylfuran	3777-70-6	1097	14.36
76	2,3,6,7-tetramethyloctane	52670-34-5		14.59
77	2,4,6-trimethyldecane	62108-27-4		15.11
78	3-hydroxy-2-methylpyran-4-one	118-71-8	1094	15.21
79	4-methylundec-1-ene	74630-39-0	1085	15.40
80	2,2,11,11-tetramethyldodecane	127204-12-0		15.83

81	Cyclooctanone	502-49-8		15.98
82	(E)-non-3-en-2-one	14309-57-0	1146	16.41
83	Pentylbenzene	538-68-1	1166	17.15
84	2,3-dimethyldecane	17312-44-6		17.57
85	Ethyl octanoate	106-32-1	1197	19.03
86	2,6-dimethylundecane	17301-23-4	1216	19.80
87	5-(hydroxymethyl)furan-2-carbaldehyde	67-47-0	1252	20.49
88	(E)-dec-2-enal	3913-81-3	1263	21.80
89	2,3,6-trimethyldecane	62238-12-4		22.00
90	2,6,11-trimethyldodecane	31295-56-4	1275	22.73
91	2-methylsulfanylpyrimidin-5-ol	4874-33-3		23.06
92	2-octylfuran	4179-38-8		23.26
93	Undecanal	112-44-7	1307	23.82
94	3-[(E)-3-methylbut-1-enyl]cyclohexene	56030-49-0		24.17
95	(2E,4E)-deca-2,4-dienal	2363-88-4	1280	24.17
96	(E)-undec-2-enal	2463-77-6	1359	26.23
97	Tetradec-1-ene	1120-36-1	1394	27.50
98	(E)-hexadec-14-enal	330207-53-9		27.51
99	Monane-1,9-diol	3937-56-2		28.15
100	(E)-octadec-5-ene	7206-21-5		31.61
101	Pentadecane	629-62-9		31.94
102	(2Z,4E)-3,7,11-trimethyldodeca-2,4-diene			34.12
103	1-nonylcyclohexene	15232-88-9		34.59
104	(Z)-tetradec-9-en-1-ol	35153-15-2		37.32
105	Pentadecan-2-one	2345-28-0	1699	37.88

RI: Retention Index; Ret. Time: Retention Time
